# Beyond Causal Explanation: Einstein’s Principle Not Reichenbach’s

**DOI:** 10.3390/e23010114

**Published:** 2021-01-16

**Authors:** Michael Silberstein, William Mark Stuckey, Timothy McDevitt

**Affiliations:** 1 Department of Philosophy, Elizabethtown College, Elizabethtown, PA 17022, USA; 2 Department of Philosophy, University of Maryland, College Park, MD 20742, USA; 3 Department of Physics, Elizabethtown College, Elizabethtown, PA 17022, USA; stuckeym@etown.edu; 4 Department of Mathematical Sciences, Elizabethtown College, Elizabethtown, PA 17022, USA; mcdevittt@etown.edu

**Keywords:** EPR correlations, relativity principle, principle explanation, Reichenbach’s Principle, retrocausality, locality, contextuality, no preferred reference frame, causal modelling, no-signalling, quantum information theory, reconstructions of quantum mechanics, Tsirelson bound, realist psi-epistemic

## Abstract

Our account provides a local, realist and fully non-causal principle explanation for EPR correlations, contextuality, no-signalling, and the Tsirelson bound. Indeed, the account herein is fully consistent with the causal structure of Minkowski spacetime. We argue that retrocausal accounts of quantum mechanics are problematic precisely because they do not fully transcend the assumption that causal or constructive explanation must always be fundamental. Unlike retrocausal accounts, our principle explanation is a complete rejection of Reichenbach’s Principle. Furthermore, we will argue that the basis for our principle account of quantum mechanics is the physical principle sought by quantum information theorists for their reconstructions of quantum mechanics. Finally, we explain why our account is both fully realist and psi-epistemic.

## 1. Introduction

There is a class of interpretations or accounts of quantum mechanics (QM) called retrocausal theories (for more historical background and comparisons of different models, see [1,2]). Such models vary wildly, and it would seem that the only thing they have in common is that the future determines the past or present as much as the past or present determines the future, at least with respect to some QM phenomena. However, many of the purveyors of retrocausal accounts do have similar motives. Namely, to show that QM does not, contrary to certain “no-go theorems,” entail non-locality, contextuality and realism about the wavefunction and QM states. Furthermore, defenders generally agree that a retrocausal account ought to be nonetheless a realist account of QM. It is for this reason that we cannot avoid delving into some detail on the question of what constitutes a realist account of QM. Furthermore, the discussion of this topic will come in handy in explaining why our account is realist and psi-epistemic.

What exactly makes an interpretation of QM a realist one is up for debate, but at the very least, it is generally believed that realist interpretations cannot be purely epistemic. For example, according to QBism, QM probabilities are not about objective reality, rather they are about updating the belief states of single epistemic agents. Healey’s pragmatist account of QM [3] and Rovelli’s relational account of QM [4] both hold that the QM state is not a description of the physical world, but only exists to generate QM probabilities. Unlike QBism, both pragmatist accounts and relational accounts of QM are relative-state theories in a sense, the difference is that in the pragmatist account a quantum state ascription is relative only to the perspective of an actual or potential agent, whereas in relational QM values are objective and relative to any physical system—information is relative information that one physical system has about another, as with a third physical system observing two other entangled systems, etc.

The point is that in relational QM, all information is purely relational and this need have nothing to do with ‘agents’ even in the neutral sense of agent in which a non-conscious A.I. might be observing and measuring outcomes in QM experiments. However, Healey is clear that a QM state can only be ascribed to an agent in the context of an experimental set-up that defines the perspective of that agent; in this respect Healey’s pragmatist QM is a kind of half-way house between QBism and relational QM. In spite of the agent-centric talk in QBism and the pragmatist account, these accounts do not require *conscious* agents. An epistemic agent could be a non-conscious machine of some sort. What makes all three of these accounts epistemic is that they all hold the QM state is not a description of the physical world, but only exists to generate QM probabilities. That is, in addition to being explicitly psi-epistemic, none of these accounts provides an ontology or what Bell called [5] “beables,” that are allegedly hiding behind the veil of the ‘observables’ and explain the phenomenology in question. The beables, such as particles, fields or waves of some sort, are supposed to tell us exactly what happens, say, between the initiation and termination of some QM experiment, such as a Bell-type experiment or twin-slit type experiment. Clearly this notion of beables presupposes what we will shortly define as a dynamical or causal explanatory bias.

It is sometimes further claimed that beables must have some metaphysical autonomy/independence and some intrinsic properties. If that is so, then relational QM fails to be a realist theory for yet another reason. As Laudisa and Rovelli put it [4]:

For RQM (relational quantum mechanics), the lesson of quantum theory is that the description of the way distinct physical systems affect each other when they interact (and not the way physical systems ‘are’) exhausts all that can be said about the physical world. The physical world must be described as a net of interacting components, where there is no meaning to ‘the state of an isolated system’, or the value of the variables of an isolated system. The state of a physical system is the net of the relations it entertains with the surrounding systems. The physical structure of the world is identified as this net of relationships.

Thus, if relational QM is true, there are no such things as beables so defined. We will return to such questions in the Discussion and Postscript wherein we will take up the topic of contextuality and realism more explicitly. Therein, we will explain why our principle account of QM is a realist, psi-epistemic account, as well as our take on beables, etc.

Finally, in perhaps the most egregious violation of realism, some of these accounts, such as relational QM, are labeled subjectivist because they allegedly entail that, at least in certain situations such as Wigner’s friend type set-ups, different observers can consistently give different accounts of the same set of events such as the outcomes of measurements. For example, in a particular Schrödinger’s Cat type set-up, even without invoking the branching structure of the Many-Worlds interpretation, observer X can report seeing a live cat and observer Y can report seeing a dead cat, allegedly the very same cat, and both can be correct without contradiction [6] (pp. 116–117). That is, such subjectivist accounts allegedly violate what is sometimes called, The Absoluteness of Observed Events [7]. For a detailed explanation of why our principle account of QM rules out such absurdities see the Postscript and [8].

Many purveyors of retrocausal models of QM are hoping to construct realist models in the sense of being housed strictly in spacetime and requiring nothing but beables such as particles, semi-classical fields or semi-classical waves. Perhaps, then, it is not surprising that most retrocausal accounts, even those that have a block universe picture firmly in mind, still insist that the best explanation for EPR correlations must be a causal explanation of some sort. The idea here being that what it means to provide a realist account of said correlations is to provide a causal account of some sort. Whatever their motivations and whatever their particular account of retrocausation might be, such theorists still adhere to some version of Reichenbach’s Principle, which states that if two events are correlated, then either there is a causal connection between the correlated events that is responsible for the correlation or there is a third event, a so-called common cause, which brings about the correlation.

When it comes to how retrocausal accounts might thwart various no-go theorems, the focus is on the statistical “measurement independence” assumption in Bell’s theorem, i.e., the assumption that outcomes in EPR-type experiments do not causally depend on any future measurement settings. If one could construct a fully time-like retrocausal account of QM that fully explained EPR correlations with no remaining fatal flaws and which reproduced the statistics of QM, then perhaps locality (what Bell calls “local causality”) could be saved. Furthermore, if one can tell such a retrocausal account, then perhaps contrary to the Kochen–Specker theorem and others like it [9], non-contextuality can be saved as well: the claim that physical properties of QM systems exist prior to and independently of the act of measurement. As Friederich and Evans put it [1]:

Retrocausality renders Kochen-Specker-type contextuality potentially explainable as a form of “causal contextuality”. If there is a backward-directed influence of the chosen measurement setting (and context) on the pre-measurement ontic state, it is no longer to be expected that the measurement process is simply uncovering an independently existing definite value for some property of the system, rather the measurement process can play a causal role in bringing about such values (the measurement process is retrocausal rather than retrodictive). Indeed, one might argue contextuality of measured values is just what one might expect when admitting retrocausal influences. As Wharton (2014: 203) puts it, “Kochen-Specker contextuality is the failure of the Independence Assumption”, i.e., the failure of measurement independence.

Finally, the idea is that if retrocausation can thwart non-locality and contextuality, then perhaps it provides the basis for a realist, psi-epistemic account of QM in spacetime alone, with counterfactual definiteness and determinate physical properties throughout the worldtube of every QM system. Of course, while many retrocausal accounts adhere to Reichenbach’s Principle in some form, the nature of the causal relation itself varies across different retrocausal explanations. However, retrocausal explanations tend to invoke one of two (or both) notions of causation. The first kind of causation is a “causal processes account,” wherein chains of events are related by causal interactions (“action-by-contact”), that involve local exchanges of a conserved quantity. Such causal influences extend through spacetime via contiguously mediated connections between local beables, as with classical fields [10]. The trick for retrocausal accounts espousing this type of causation is to thwart non-locality (faster-than-light causal connections between space-like separated events, i.e., “spooky actions at a distance” [11] (p. 158)), by telling a story whereby such causal processes are purely time-like. Such causal processes are often described as making a time-like “zig-zag” pattern in spacetime between the two space-like separated detectors in a standard EPR-type setup [12,13,14]. We should note that some such accounts are still realist about the QM wavefunction or at least about semi-classical waves in spacetime (the general idea here is that wavefunctions evolve both forwards and backwards in time), but not all [15,16].

The second type of causation is called the “interventionist” or “manipulability” account of causation [17]. The central idea is that X is a cause of Y if and only if manipulating X is an effective means of indirectly manipulating Y. According to retrocausal accounts of QM espousing an interventionist account of causation, manipulating the setting of a measurement apparatus now can be an effective means of manipulating aspects of the past. The formal machinery of causal modelling has the interventionist account of causality as its foundation [18].

Price and Wharton, two key defenders of retrocausal accounts of QM, embrace a subset of interventionism known as the “agent” or “perspectivalism” account of causation [19,20,21]. On this view, causal relations are relations that can be used for control or manipulation, from the perspective of the agent in question of course. This is an understandably appealing notion of causation for those such as Price and Wharton who espouse a block universe picture, wherein causation talk cannot possibly be about changing or bringing about events (past, present or future) in any robust sense of those terms. In our language [22], agent causation focuses on the “ant’s-eye” view of explanation from within the block universe, as opposed to the “God’s-eye” view that would seek a purely objective explanation for EPR correlations external to a perspective from within the block universe, an explanation that transcends and subsumes perspectival causation, such as conservation laws.

In addition to the specific problems faced by particular retrocausal models, the consensus is that, as of yet, no realist retrocausal account manages to successfully save locality, non-contextuality and a psi-epistemic account of the wavefunction [1]. The most general concern however is that retrocausal accounts fail to provide a robust or coherent causal explanation of EPR correlations and contextuality. The most general form of this concern is that, at least from the God’s-eye point of view, the very idea of retrocausation in a block universe makes very little physical or explanatory sense [13,23,24]. Many (though not all [13]) believe that retrocausation demands a block universe. This is because it is hard to see how the future or future boundary conditions could cause anything or participate in any type of explanation of EPR correlations, if the future does not exist. Yet, when we think of the block universe from the God’s-eye point of view, it is clear that causation cannot be about bringing new events into being that did not formerly exist, because from a God’s-eye point of view it is all just ‘there’, including EPR-experiments from initiation (source) to termination (detector). The very idea of “causality flowing backwards in time” as with the “causal processes account,” simply seems superfluous or redundant in such a world. For example, as Cramer says himself, the backwards-causal elements of his transactional interpretation are “only a pedagogical convention,” and that in fact “the process is atemporal” [25] (p. 661). But the idea of an “atemporal process” seems like a non-sequitur. In a block universe, why bother trying to add some new mechanism (such as waves from the future) to account for how information from the future got to the emission event in the past? Again, from a God’s-eye point of view the relevant information at every point in the “process” from source to detector, is all just ‘there’. Aside from thwarting non-locality, how is this backward brand of causation any better at saving constructive or commonsense notions of causation than “instantaneous causation” between space-like separated events?

Those who advocate for an interventionist or perspectivalist account of causation would argue that such an account of causation still makes sense even in a block universe. However, there are problems with this account of causation as well. As Friederich and Evans note [1]:

Two of the more significant assumptions are (i) the causal Markov condition, which ensures that every statistical dependence in the data results in a causal dependence in the model—essentially a formalization of Reichenbach’s common cause principle—and (ii) faithfulness, which ensures that every statistical independence implies a causal independence, or no causal independence is the result of a fine-tuning of the model.

It has long been recognized (Butterfield 1992; Hausman 1999; Hausman and Woodward 1999) that quantum correlations force one to give up at least one of the assumptions usually made in the causal modeling framework. Wood and Spekkens (2015) argue that any causal model purporting to causally explain the observed quantum correlations must be fine-tuned (i.e., must violate the faithfulness assumption). More precisely, according to them, since the observed statistical independences in an entangled bipartite quantum system imply no signalling between the parties, when it is then assumed that every statistical independence implies a causal independence (which is what faithfulness dictates), it must be inferred that there can be no (direct or mediated) causal link between the parties. Since there is an observed statistical dependence between the outcomes of measurements on the bipartite system, we can no longer account for this dependence with a causal link unless this link is fine tuned to ensure that the no-signalling independences still hold. There is thus a fundamental tension between the observed quantum correlations and the no-signalling requirement, the faithfulness assumption and the possibility of a causal explanation.

We would say that even if interventionist and causal modelling accounts of causation could be applied to EPR correlations with nothing like the preceding concerns, there is still little reason to find such explanations deeply satisfying. Is there really no more fundamental and objective, God’s-eye explanation for EPR correlations that transcends and subsumes perspectival causation? Such interventionist explanations strike us as too cheap and easy, and not very deep from the perspective of fundamental physics.

In addition to the foregoing concerns, there are recent no-go theorems which allege that no account of QM can escape contextuality, because it is necessary to reproduce the observed statistics of quantum theory [9]. More recent no-go theorems allege to show that not even accounts that give up measurement independence, such as retrocausal, superdeterministic or even non-local models of QM can escape one or another strong form of contextuality, going so far as to claim that what is contextual is not just the QM state, but many other features of QM, such as what counts as a system, dynamical law and boundary conditions [26]. Going even further, Bong et al. [7] allege to provide a new and more powerful no-go theorem that we must give up at least one of the following assumptions [7]:

- Assumption 1 (Absoluteness of Observed Events-AOE): An observed event is a real single event, and not relative to anything or anyone (realism and non-contextuality).
- Assumption 2 (No-Superdeterminism-NSD): Any set of events on a space-like hypersurface is uncorrelated with any set of freely chosen actions subsequent to that space-like hypersurface.
- Assumption 3 (Locality-L): The probability of an observable event *e* is unchanged by conditioning on a space- like-separated free choice *z*, even if it is already conditioned on other events not in the future light-cone of *z*.
- Assumption 4 (The completeness of QM-COMP): QM unmodified applies to any and all macroscopic measuring devices including human observers.

Based on these and similar results, other people make even stronger claims about what the new no-go theorems and experiments show. For example, Renner claims the new theorems are telling us that QM needs to be replaced [27]. Herein, we do not address any of these new no-go theorems directly, we simply note that if these no-go results stand and if the primary goal for most retrocausal accounts is to save locality, non-contextuality, psi-epistemic, and something like classical realism, things are looking increasingly grim.

One might think that advocates of retrocausal accounts would take heart from the Bong et al. results [7], because at least it leaves superdeterminism as an option, and superdeterminism is one way to give up measurement independence. While retrocausal accounts are often labeled as superdeterministic it is important to see that they are different. Technically speaking, in a superdeterministic world, measurement independence is violated via a past common cause, for example, a common cause of one’s choice of measurements and the particle spin properties in the case of Bell correlations. Thus, superdeterminism is a conspiratorial theory with only past-to-future causation. It is true that superdeterminism entails that experimenters are not free to choose what to measure without being influenced by events in the distant past, and thus it does give up measurement independence, however, it does so in a particularly spooky way. Superdeterminism forces us to accept some very special conditions at the big bang as a brute fact or seek some sort of physically acceptable explanation for those initial conditions, that is presumably not some sort of supernatural conspiracy. While there are those who defend superdeterminism [28], most retrocausal theorists want to avoid it for the foregoing reasons.

In our book, we noted that most people are predisposed to think dynamically/causally because our perceptions are formed in a time-evolved fashion. Therefore, we want to understand/explain what we experience dynamically/causally [22]. We call this the dynamical or causal explanatory bias. It is not surprising that most people, including philosophers and physicists, have this bias. What is maybe somewhat surprising is that, as we have just seen, even retrocausal thinkers and blockworlders share this bias, i.e., they embrace the causal processes model and/or the perspectival causation model of explanation as fundamental or essential in some way. Take the following admonition from Price and Wharton [29] (p. 123):

In putting future and past on an equal footing, this kind of approach is different in spirit from (and quite possibly formally incompatible with) a more familiar style of physics: one in which the past continually generates the future, like a computer running through the steps in an algorithm. However, our usual preference for the computer-like model may simply reflect an anthropocentric bias. It is a good model for creatures like us, who acquire knowledge sequentially, past to future, and hence find it useful to update their predictions in the same way. But there is no guarantee that the principles on which the universe is constructed are of the sort that happens to be useful to creatures in our particular situation. Physics has certainly overcome such biases before—the Earth isn’t the center of the universe, our sun is just one of many, there is no preferred frame of reference. Now, perhaps there’s one further anthropocentric attitude that needs to go: the idea that the universe is as “in the dark” about the future as we are ourselves.

We share their sentiment, but even leading retrocausalists Price and Wharton are committed to causal explanations of EPR correlations of either the causal processes account or the interventionist/perspectivalist account [1].

As Friederich and Evans suggest and we concur, aside from ourselves, Wharton and Price have come the farthest in moving away from the dynamical/causal explanatory bias. Here is how they describe Wharton’s view [1]:

The account is a retrocausal picture based on Hamilton’s principle and the symmetric constraint of both initial and final boundary conditions to construct equations of motion from a Lagrangian, and is a natural setting for a perspectival interventionist account of causality. Wharton treats external measurements as physical constraints imposed on a system in the same way that boundary constraints are imposed on the action integral of Hamilton’s principle; the final measurement does not simply reveal preexisting values of the parameters, but constrains those values (just as the initial boundary condition would). Wharton’s model has been described as an “all-at-once” approach, since the dynamics of physical systems between an initial and final boundary emerges en bloc as the solution to a two-time boundary value problem.

On this interpretation, one considers reality exclusively between two temporal boundaries as being described by a classical field ϕ that is a solution to the Klein-Gordon equation: specification of field values at both an initial and final boundary (as opposed to field values and their rate of change at only the initial boundary) constrains the field solutions between the boundaries.

While Wharton’s “all-at-once” or “Lagrangian” model goes some way toward relinquishing said bias, as noted above, it still falls within the causal processes account and the interventionist/perspectivalist account of causal explanation. After all, one goal of Wharton’s retrocausal Lagrangian method is to “fill in” the classical field between initial (source) and final boundary conditions (detector). The Lagrangian method begins describing the space of possible space-time trajectories of the system between two boundary conditions, and then a least action principle such as the path of least time—a global constraint—is used to fix which of these trajectories is actual. More recently Wharton has focused on constructing ignorance-based interpretations of the path integral formalism [15]. The bottom line is that Reichenbach’s Principle is still the “axiom of choice” even when it comes to “all-at-once” or “Lagrangian” models of EPR correlations.

As we said, there is as of yet no retrocausal model that recovers the statistics of QM and also saves locality, non-contextuality, psi-epistemic, and classical realism. Over the years we have argued that the problem with retrocausal accounts is that they do not go far enough in relinquishing their dynamical/causal explanatory bias [2,22,23]. It is not enough for such models to be temporally symmetric, but rather when it comes to EPR correlations we ought to cast off the dynamical and causal mode of explanation completely, in favor of explanation a la adynamical and acausal global constraints (AGC). For example, we need not worry about the traces particles carry of their dynamical interactions, whether past or future. For us, the explanatory requirement of a particle traveling along a determinate trajectory is a holdover from the dynamical/causal bias as exemplified by the causal processes account of explanation. Rather, we should seek a quantum event or action connecting the source and detector. Lewis gets us exactly right in his description of our view as follows [30] (p. 187):

The idea is to give up the search for forward-acting and backward- acting dynamical laws that can somehow “fit together” in a consistent way to yield quantum phenomena. Rather, we derive quantum phenomena directly from a global constraint, without any appeal to the dynamical evolution of particle properties or wave functions.

We are primarily inspired to construct an AGC-based physical model by the belief that what both relativity and QM are trying to tell us, is that sometimes AGC-type explanations and contextuality are more fundamental than causal or dynamical explanation. In terms of our specific motivations as regards QM, we are primarily interested in constructing a realist, psi-epistemic, local account of QM, that fully comports with a realist view of Minkowski spacetime. That is, we do not believe in Hilbert space/wavefunction realism and we do not believe there is any action-at-a-distance that violates the causal structure (the light-cone structure) of special relativity (SR). Obviously, for us, a realist account of QM need not involve causal or dynamical explanation; we are perhaps alone in fully rejecting Reichenbach’s Principle for such cases. However, a realist account of QM ought to explain why EPR correlations exist in Minkowski spacetime. Equally obvious, if we are right in fully rejecting Reichenbach’s Principle and the dynamical/causal explanatory bias in QM, then all of these debates about the various problems of retrocausal models are a red herring.

It is one thing to posit explanations in terms of adynamical and acausal global constraints, but it is quite another to cook up a specific model. In our book [22], we posited an ontology of four-dimensional entities that do not move or change but make up the block universe. We also posited an AGC mode of explanation for EPR correlations, among other things, that uses the initial and final states of the system, plus the AGC, to provide a spatiotemporal explanation of said correlations. However, even those somewhat open to our relatively “radical” project of completely jettisoning Reichenbach’s Principle and the dynamical and causal explanatory bias behind it, found our specific account formally daunting, vague, and insufficiently precise. Lewis expresses a common concern [30] (p. 188):

But the way is not altogether clear. Classical adynamical techniques, such as least-action calculations, output a determinate trajectory between two points. But quantum adynamical techniques, such as Feynman’s path-integral calculation, output a probability value based on a sum over all possible trajectories between the two points. Which trajectory does the particle take? And what does the probability represent?

Silberstein, Stuckey and McDevitt take this situation to point to direct action between the source and the detector: But what of the probability? A global constraint that rules out non-parabolic baseball trajectories is easy to comprehend. But it is harder to figure out how to understand a probabilistic global constraint. What is constrained, exactly? The frequency of this kind of event?

Take the following even more telling reaction to our book [31] (p. 344):

I am not sold that the adynamical picture is truly explanatory. Philosophers of science have proposed objective accounts of explanation, but they all recognize there’s a strong sense in which explanation is ‘explanation for us,’ and any account should capture our intuition that explanation is fundamentally dynamical. This is connected with causation: intuitively, we explain an event because we find its causes; causes happen before their effects and ‘bring them about.’ An empiricist will be skeptical of causation, like presumably SSM. However, as is well known, one can dispense of causation and propose models of explanations in which laws of nature and unification of phenomena play an important role. Should I think of SSM’s adynamical view in this sense? Or should I connect their view with the distinction between constructive and principle theories, proposed by Einstein (1919)? According to Einstein, principle theories (like thermodynamics) are formulated in terms of principles that systematize the phenomena; so that one has explained an event if it follows from the principles. In contrast, in a constructive theory (as kinetic theory) a phenomenon is explained when it fits into the ‘mechanical’ model of the theory. Should I understand SSM’s view as a principle theory? (But if so, which are the principles?).

In the preceding passage, Allori beautifully expresses the aforementioned recalcitrance of the dynamical and causal explanatory bias. However, more importantly, Allori suggests another way to conceive of our project in terms of providing a principle versus constructive account of QM generally and EPR correlations specifically. This is precisely what we have done in recent subsequent work [32,33,34], and we will expand upon those results herein.

Finally, our principle account of QM introduced in Section 3 shows a profound unity between QM and SR that is generally unappreciated, especially since by “QM” we are referring to non-relativistic quantum mechanics. We begin in Section 2, with an overview of principle versus constructive explanation in general and the recent history of that debate within QM itself. We present our principle account of QM in Section 3, showing how it resolves a number of QM mysteries. We conclude with Section 4, where we defend our principle account of QM and its obvious implication for causality in physics. In the Postscript we will return to the question of why our principle account is both realist and psi-epistemic, the place of contextuality, etc.

## 2. Principle Versus Constructive Explanation

Here, we begin with some background needed to appreciate our explanatory project. As we will see, some theorists in QM, such as Fuchs and Hardy, point to the postulates of SR as an example of what quantum information theorists (QIT) seek for QM, and SR is a “principle theory” [35]. That is, the postulates of SR are constraints without a corresponding “constructive” or causal explanation. Here, Einstein explains the difference between the two [36]:

We can distinguish various kinds of theories in physics. Most of them are constructive. They attempt to build up a picture of the more complex phenomena out of the materials of a relatively simple formal scheme from which they start out. [Statistical mechanics is an example.]...

Along with this most important class of theories there exists a second, which I will call “principle-theories.” These employ the analytic, not the synthetic, method. The elements which form their basis and starting point are not hypothetically constructed but empirically discovered ones, general characteristics of natural processes, principles that give rise to mathematically formulated criteria which the separate processes or the theoretical representations of them have to satisfy. [Thermodynamics is an example.]...

The advantages of the constructive theory are completeness, adaptability, and clearness, those of the principle theory are logical perfection and security of the foundations. The theory of relativity belongs to the latter class.

Concerning his decision to produce a principle theory instead of a constructive theory of SR, Einstein writes [37] (pp. 51–52):

By and by I despaired of the possibility of discovering the true laws by means of constructive efforts based on known facts. The longer and the more despairingly I tried, the more I came to the conviction that only the discovery of a universal formal principle could lead us to assured results.

That is, “there is no mention in relativity of exactly *how* clocks slow, or *why* meter sticks shrink” (no “constructive efforts”), nonetheless the principles of SR are so compelling that “physicists always seem so sure about the particular theory of Special Relativity, when so many others have been superseded in the meantime” [38].

Today, we find ourselves in a similar situation with QM. That is, 85 years after the famous EPR paper [39] we still have no consensus constructive account of QM. This prompted Smolin to write [40] (p. 227):

So, my conclusion is that we need to back off from our models, postpone conjectures about constituents, and begin talking about principles.

Fuchs writes [41] (p. 285):

Compare [quantum mechanics] to one of our other great physical theories, special relativity. One could make the statement of it in terms of some very crisp and clear physical principles: The speed of light is constant in all inertial frames, and the laws of physics are the same in all inertial frames. And it struck me that if we couldn’t take the structure of quantum theory and change it from this very overt mathematical speak..., then the debate would go on forever and ever. And it seemed like a worthwhile exercise to try to reduce the mathematical structure of quantum mechanics to some crisp physical statements.

And, Hardy writes [42]:

The standard axioms of [quantum theory] are rather ad hoc. Where does this structure come from? Can we write down natural axioms, principles, laws, or postulates from which we can derive this structure? Compare with the Lorentz transformations and Einstein’s two postulates for special relativity. Or compare with Kepler’s Laws and Newton’s Laws. The standard axioms of quantum theory look rather ad hoc like the Lorentz transformations or Kepler’s laws. Can we find a natural set of postulates for quantum theory that are akin to Einstein’s or Newton’s laws?

Along those lines, QIT have produced several reconstructions of QM, but they are so far not compelling. Dakic and Brukner write [43]:

The vast majority of attempts to find physical principles behind quantum theory either fail to single out the theory uniquely or are based on highly abstract mathematical assumptions without an immediate physical meaning (e.g., [18]). ...

While [the instrumentalist] reconstructions are based on a short set of simple axioms, they still partially use mathematical language in their formulation. ...

It is clear from the previous discussion that the question on basis of which physical principles quantum theory can be separated from the multitude of possible generalized probability theories is still open.

Another problem with the reconstructions of QIT is noted by Van Camp [44]:

However, nothing additional has been shown to be incorporated into an information-theoretic reformulation of QM beyond what is contained in QM itself. It is hard to see how it could offer more unification of the phenomena than QM already does since they are equivalent, and so it is not offering any explanatory value on this front.

Moreover, Fuchs quotes Wheeler, “If one really understood the central point and its necessity in the construction of the world, one ought to state it in one clear, simple sentence” [41] (p. 302). Asked if he had such a sentence, Fuchs responded, “No, that’s my big failure at this point” [41] (p. 302). Herein, we answer the desideratum of QIT explicitly by showing how the relativity principle, aka “no preferred reference frame” (NPRF), is the physical principle corresponding to the reconstructions of QM, just as it is for the Lorentz transformations of SR.

Our claim about principle explanation being “fundamental” deserves some unpacking. Obviously, the question of what makes an explanation relatively “fundamental” is multifaceted (i.e., there are multiple senses of “fundamental”) and value laden. Our claim about the fundamentality of principle explanation in this case amounts to this:

1. The principle explanation on offer is compatible with a number of different constructive interpretations of QM and will not be nullified or made redundant by any of them, just as with SR and thermodynamics. Thus, the principle explanation on offer is fundamental in the sense that it is more general, universal and autonomous than any particular constructive explanation or interpretation.
2. As with the case of SR, the principle explanation on offer suggests the possibility that there will never be and need never be, any constructive theory to underwrite it or subsume it. Dynamical and causal bias aside, there is no reason to rule out this possibility a priori, and SR looks to be such a case already. Thus, the principle explanation herein would be fundamental in the sense that it does not even in principle reduce to some constructive theory or explanation.

One might ask, does our principle explanation at least rule out any particular constructive interpretations of QM, or make them redundant? To which we would reply, does thermodynamics rule out or make redundant statistical mechanics or particular alternative microphysical theories? Is the converse true? Does SR rule out or make redundant alternative constructive accounts about phenomena such as Lorentz contractions? Is the converse true? Regardless of one’s larger metaphysical commitments, the consensus answer to all these questions is in the negative. Let us return the focus to QM. The Lagrangian and Hamiltonian formulations of QM do not rule each other out or make one another redundant. And this claim is perfectly compatible with a psi-epistemic account of the wavefunction (see the Postscript for more details). What a principle account does is constrain constructive theories, beyond the constraints in question, it does not necessarily rule them out or make them redundant. However, a principle theory can make constructive accounts redundant, as with the case of SR and the luminiferous ether. But as we note in the Discussion, while people have abandoned theories of the luminiferous ether, some still insist there must be an underlying constructive explanation for relativistic effects such as length contraction. We disagree, but we have no way of ruling out this possibility in principle. However, as we noted above in point 1, even if there is such a constructive explanation forthcoming, SR would still be fundamental in the sense of generality, universality and autonomy.

Regardless of where one stands on these matters, there is no denying the fact that SR, with its principle explanation, has led to profound advancements in physics and we are offering a similar possibility for QM. Of course, this is not to say that our principle account creates no tension whatsoever with certain constructive accounts of, say, EPR correlations. If one is willing to accept the possibility that a principle explanation such as ours will never be reduced or underwritten by a causal or dynamical constructive account, then we have a completely local, adynamical and acausal explanation for EPR correlations that dissolves any tension between QM and SR. In short, if we are right and our principle explanation is fundamental as characterized by point 2 above, then we simply do not need constructive non-local accounts of EPR correlations, such as Bohmian mechanics and spontaneous collapse accounts. [See the Postscript for our broader interpretative commitments regarding QM.]

However, our own view aside, for those who hold that fundamental explanation must be constructive and realist in Einstein’s sense of those words, none of the mainstream interpretations neatly fit the bill. Not only do most interpretations entail some form of QM holism, contextuality, and/or non-locality, the remainder invoke priority monism and/or multiple branches or outcomes. The problem with attempting a constructive account of QM is, as articulated by Van Camp, “Constructive interpretations are attempted, but they are not unequivocally constructive in any traditional sense” [44]. Thus, he states [44]:

The interpretive work that must be done is less in coming up with a constructive theory and thereby explaining puzzling quantum phenomena, but more in explaining why the interpretation counts as explanatory at all given that it must sacrifice some key aspect of the traditional understanding of causal-mechanical explanation.

If statistical mechanics is the paradigm example of constructive explanation, then it is hard to imagine Einstein would approve of any mainstream interpretations of QM.

Let us also note again that contrary to certain others, we are arguing that principle explanation need not ever be discharged by a constructive explanation or interpretation–causal or otherwise in SR [45,46,47,48] or in QM [49]. For example, our principle explanation avoids the complaints about Bub’s proposed principle explanation of QM leveled by Felline [49]. That is, the principle being posited herein does not require a solution to the measurement problem nor again does it necessarily beg for a constructive counterpart.

**Figure 1:**
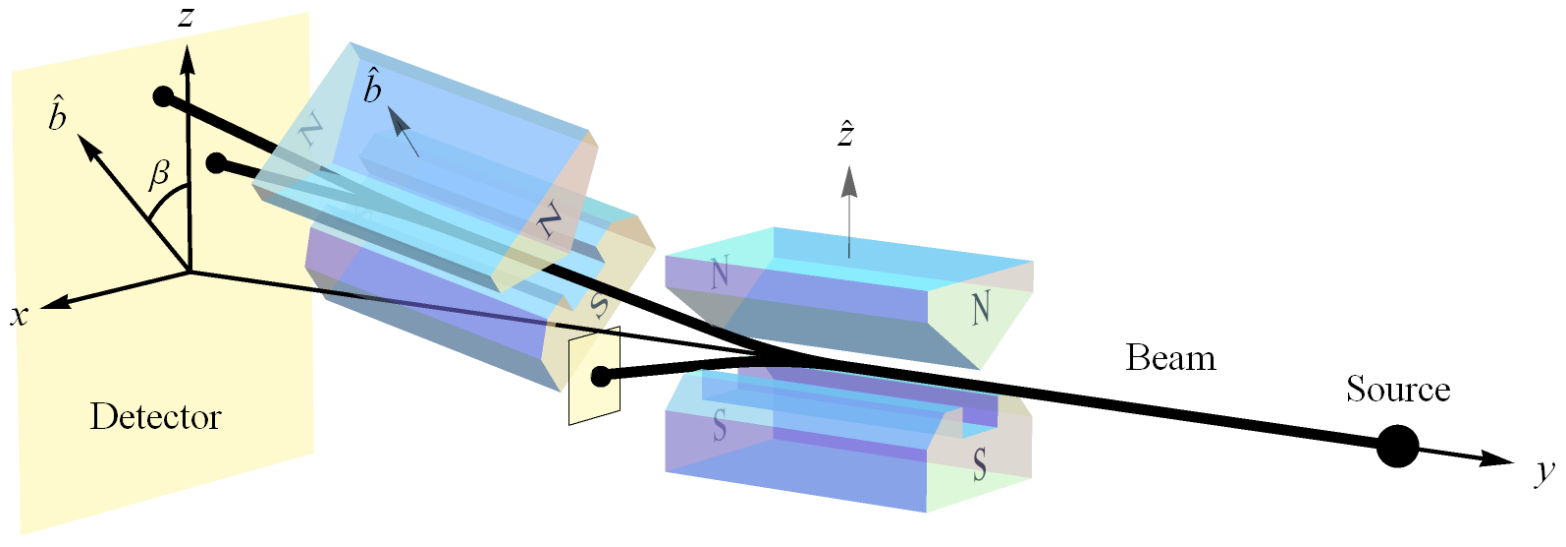
A pair of Stern–Gerlach (SG) spin measurements each showing the two possible outcomes, up ($+\frac{\hbar }{2}$) and down ($-\frac{\hbar }{2}$) or +1 and −1, for short. In this set up, the first SG magnets (oriented at $\hat{z}$) are being used to produce an initial state |ψ〉=|u〉 for measurement by the second SG magnets (oriented at $\hat{b}$). An important point to note here is that the classical analysis predicts all possible deflections between the target points on the detector, not just the two that are observed. The difference between the classical prediction and the quantum reality uniquely distinguishes the quantum joint distribution from the classical joint distribution for the Bell spin states [50].

To be specific, we extend NPRF from its application to the measurement of the speed of light *c* to include the measurement of another fundamental constant of nature, Planck’s constant *h*. As Weinberg has noted, measuring an electron’s spin via Stern–Gerlach (SG) magnets constitutes the measurement of “a universal constant of nature, Planck’s constant” [51] (Figure 1). Thus, if NPRF applies equally here, everyone must measure the same value for Planck’s constant *h* regardless of their SG magnet orientations relative to the source, which is an “empirically discovered” fact just like the light postulate. By “relative to the source,” we mean relative to the plane perpendicular to the particle beam (Figure 1). In this case, the spin outcomes $\pm \frac{\hbar }{2}$ represent fundamental (indivisible) units of information per Dakic and Brukner’s first axiom in their reconstruction of quantum theory, “An elementary system has the information carrying capacity of at most one bit” [43]. Therefore, the different SG magnet orientations relative to the source constitute different “reference frames” in QM, just as the different velocities relative to the source constitute different “reference frames” in SR.

**Figure 2:**
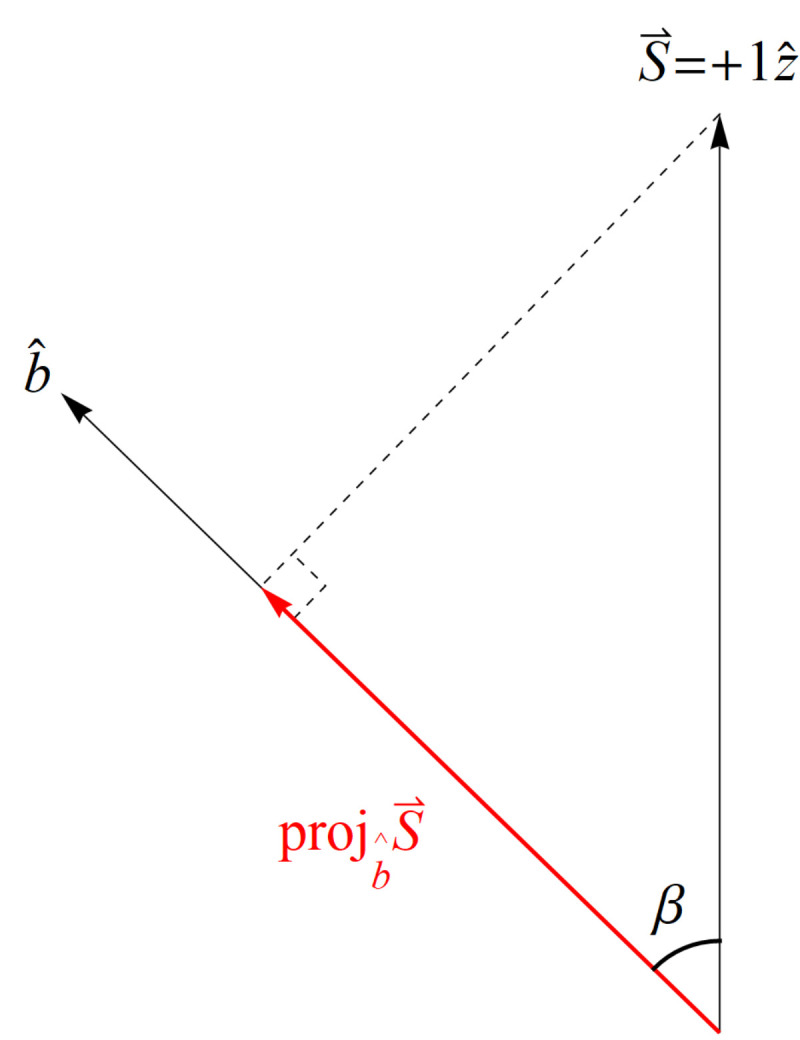
The spin angular momentum of Bob’s particle $\vec{S}$ projected along his measurement direction $\hat{b}$. This does *not* happen with spin angular momentum due to no preferred reference frame (NPRF).

To make the analogy more explicit, one could have employed NPRF to predict the light postulate as soon as Maxwell showed electromagnetic radiation propagates at $c=\frac{1}{\sqrt{\mu_{o}\epsilon_{o}}}$. All they would have had to do is extend the relativity principle from mechanics to electromagnetism. However, given the understanding of waves at the time, everyone rather began searching for a propagation medium, i.e., the luminiferous ether. Likewise, one could have employed NPRF to predict spin angular momentum as soon as Planck published his wavelength distribution function for blackbody radiation. All they would have had to do is extend the relativity principle from mechanics and electromagnetism to blackbody radiation. However, given the understanding of angular momentum and magnetic moments at the time, Stern and Gerlach rather expected to see their silver atoms deflected in a continuum distribution after passing through their magnets (Figure 1). In other words, they discovered spin angular momentum when they were simply looking for angular momentum. However, had they noticed that their measurement constituted a measurement of Planck’s constant (with its dimension of angular momentum), they could have employed NPRF to predict the spin outcome with its qubit Hilbert space structure (Figure 1 and Figure 2) and its ineluctably probabilistic nature, as we detail in Section 3.

We can certainly imagine a world where NPRF did not apply to *c* and *h*. In the former case, *c* would only be measured in the “hidden” preferred frame of the luminiferous ether. In that case, the kinematic and causal structure of Minkowski spacetime would not obtain. In the latter case, *h* would only be measured in the “hidden” preferred frame of the orientation of the electron’s angular momentum. In that case, the non-Boolean qubit Hilbert space structure would not obtain. Bub and Pitowski have pointed out the analogy between Minkowski spacetime and Hilbert space [52,53,54] in an attempt to explain EPR correlations. Bub sums it up nicely [55]:

Hilbert space as a projective geometry (i.e., the subspace structure of Hilbert space) represents the structure of the space of possibilities and determines the kinematic part of quantum mechanics. ... The possibility space is a non-Boolean space in which there are built-in, structural probabilistic constraints on correlations between events (associated with the angles between the rays representing extremal events) – just as in special relativity the geometry of Minkowski space-time represents spatio-temporal constraints on events. These are kinematic, i.e., pre-dynamic, objective probabilistic or information-theoretic constraints on events to which a quantum dynamics of matter and fields conforms, through its symmetries, just as the structure of Minkowski space-time imposes spatio-temporal kinematic constraints on events to which a relativistic dynamics conforms.

But as a mere analogy, it lacks explanatory power. Herein we complete their explanatory project by showing why both aspects of their analogy follow from a common principle, NPRF.

Since QIT reconstructions of QM are based fundamentally in composite fashion on the qubit [42,43], the “very crisp and clear physical principle” of NPRF underwriting the qubit Hilbert space structure therefore underwrites the QIT reconstructions of QM. This advances QM from a mere operational theory to a proper principle theory, at least Hardy and Dakic and Brukner’s reconstructions thereof. Indeed, NPRF as the physical principle behind the reconstructions of QM provides more than a mere analogy between the Lorentz transformations and the postulates of SR. That is, NPRF is to the QIT reconstructions of QM as NPRF is to the Lorentz transformations of SR. And, the fundamental transformation for the qubit at the foundation of QIT reconstructions is SO(3) [43], so we see that SO(3) and the Lorentz boosts close as a transformation group (the restricted Lorentz group) relating different reference frames in QM and SR, respectively. This also motivated Dakic and Brukner’s axiom 3, which was “assumed alone for the purposes that the set of transformations builds a group structure” [43].

Essentially, we resolve the primary problem with QIT attempts to “find physical principles behind quantum theory,” i.e., that they “either fail to single out the theory uniquely or are based on highly abstract mathematical assumptions without an immediate physical meaning,” by explaining the qubit Hilbert space structure using constraints on QM processes in spacetime, i.e., “average-only” projection and “average-only” conservation per NPRF, rather than the converse. Thus, analogous with the structure of spacetime in SR, our principle account of QM shows how the qubit Hilbert space structure follows from the relativity principle in spacetime, as opposed to the converse.

At the outset of Section 3, we articulate the connection between NPRF and the qubit Hilbert space structure, quantum contextuality, and the ineluctably probabilistic nature of QM. We then extrapolate this result to biparite entangled qubit systems to show why the mystery of Bell state entanglement results from conservation per NPRF in Section 3.1 and Section 3.2. This will make it clear how conservation per NPRF rules out what Dakic and Brukner call “mirror quantum mechanics” in their reconstruction of QM.

## 3. QM from NPRF Whence Bell State Entanglement

We will refer explicitly to SG spin measurements for visualization purposes, but this can be understood to represent any measurement with a binary outcome in the symmetry plane. The only other outcome pair would be perpendicular to the symmetry plane, as in “*V*” (+1) or “*H*” (−1) outcomes with photons and polarizers, in which case one thinks of “intensity of the transmitted beam” rather than “projection of the transmitted vector” [32]. The binary outcome still represents the invariant measure of the fundamental unit of action *h* with respect to the SO(3) transformations between QM reference frames, as in all quantum exchanges [34]. Again, SO(3) with Lorentz boosts then complete the restricted Lorentz transformation group between reference frames. As shown explicitly by Dakic and Brukner [43], the SO(3) transformation group uniquely identifies the fundamental probability structure of QM amid those of classical probability theory and higher-dimensional generalized probability theories.

**Figure 3:**
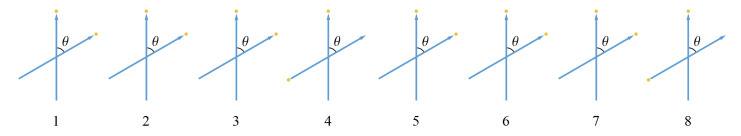
A spatiotemporal ensemble of 8 SG measurement trials. The blue arrows depict SG magnet orientations and the yellow dots represent the two possible measurement outcomes for each trial, up (located at arrow tip) or down (located at bottom of arrow). The vertical arrow can represent an initial state |ψ〉=|u〉 in which case the other arrow represents an SG measurement at $\theta =60^{\circ }$ of |ψ〉. In that case, we see that the average of the ±1 outcomes equals the projection of the initial spin angular momentum vector $\vec{S}=+1\hat{z}$ in the measurement direction $\hat{b}$, i.e., $\vec{S}\cdot \hat{b}=\cos\left(60^{\circ }\right)=\frac{1}{2}$. The figure can also depict two SG measurements of a spin triplet state showing Bob’s(Alice’s) outcomes corresponding to Alice’s(Bob’s) +1 outcomes when $\theta =60^{\circ }$. For the triplet state measurements, spin angular momentum is not conserved in any given trial, because there are two different measurements being made, i.e., outcomes are in two different reference frames, but it is conserved on average for all 8 trials (six up outcomes and two down outcomes average to $\cos\left(60^{\circ }\right)=\frac{1}{2}$). It is impossible for spin angular momentum to be conserved explicitly in each trial since the measurement outcomes are binary (quantum) with values of +1 (up) or −1 (down) per NPRF. The “SO(3) conservation” at work here does not assume Alice and Bob’s measured values of spin angular momentum are mere components of some hidden angular momentum (Figure 2). That is, the measured values of spin angular momentum *are* the angular momenta contributing to this “SO(3) conservation.”

If we create a preparation state oriented along the positive *z* axis as in Figure 1, i.e., |ψ〉=|u〉, our spin angular momentum is $\vec{S}=+1\hat{z}$ (in units of $\frac{\hbar }{2}=1$). Now proceed to make a measurement with the SG magnets oriented at $\hat{b}$ making an angle β with respect to $\hat{z}$ (Figure 1). According to classical physics, we expect to measure $\vec{S}\cdot \hat{b}=\cos\left(\beta \right)$ (Figure 2), but we cannot measure anything other than ±1 due to NPRF (contra the prediction by classical physics), so we see that NPRF answers Wheeler’s “Really Big Question,” “Why the quantum? [56,57]” in “one clear, simple sentence” to convey “the central point and its necessity in the construction of the world.” As a consequence, we can only recover cos(β) *on average* (Figure 3), i.e., NPRF dictates “average-only” projection

(1) (+1)P(+1∣β)+(−1)P(−1∣β)=cos(β)

Solving simultaneously with P(+1∣β)+P(−1∣β)=1, we find that

(2) $$P\left(+1|\beta \right)=\cos^{2}\left(\frac{\beta }{2}\right)$$

and

(3) $$P\left(-1|\beta \right)=\sin^{2}\left(\frac{\beta }{2}\right)$$

When talking about the longitudinal outcomes [58] (“click” or “no click”), we have

(4) $$P\left(V|\beta \right)=\cos^{2}\left(\beta \right)$$

and

(5) $$P\left(H|\beta \right)=\sin^{2}\left(\beta \right)$$

so that our average outcome at β (orientation of polarizer with respect to initial polarization state) is given by

(6) $$\left(+1\right)\cos^{2}\left(\beta \right)+\left(-1\right)\sin^{2}\left(\beta \right)=\cos^{2}\left(\beta \right)-\sin^{2}\left(\beta \right)$$

This is the naively expected Malus law per classical physics for the intensity of electromagnetic radiation transmitted through a polarizer if “pass” is +1 and “no pass” is −1 (instead of 0). As with the transverse mode NPRF rules out “fractional outcomes,” again contra the prediction by classical physics, so the classical result obtains only on average when β≠0. This explains the ineluctably probabilistic nature of QM, as pointed out by Mermin [59]:

Quantum mechanics is, after all, the first physical theory in which probability is explicitly not a way of dealing with ignorance of the precise values of existing quantities.

So, we have answered Lewis’ question cited earlier, “What does the probability represent?” [30] (p. 188). Of course, these “average-only” results due to “no fractional outcomes per NPRF” hold precisely for the qubit Hilbert space structure of QM.

We ask for the reader’s indulgence while we explicitly review how the qubit Hilbert space structure represented by the Pauli spin matrices evidences the relationship between quantum contextuality and NPRF implicitly. In the eigenbasis of $\sigma_{z}$ the Pauli spin matrices are

σx=0001100,σy=00−ii0,andσz=1000−1.

where $i=\sqrt{-1}$. All spin matrices have the same ±1 eigenvalues (measurement outcomes), which reflects the fact that there are no fractional outcomes per NPRF. We denote the corresponding eigenvectors (eigenstates) as |u〉 and |d〉 for spin up (+1) and spin down (−1), respectively. Using the Pauli spin matrices supra with $|u\rangle =\left(\begin{array}{c} 1\\ 0 \end{array}\right)$ and $|d\rangle =\left(\begin{array}{c} 0\\ 1 \end{array}\right)$, we see that $\sigma_{z}|u\rangle =|u\rangle$, $\sigma_{z}|d\rangle =-|d\rangle$, $\sigma_{x}|u\rangle =|d\rangle$, $\sigma_{x}|d\rangle =|u\rangle$, $\sigma_{y}|u\rangle =i|d\rangle$, and $\sigma_{y}|d\rangle =-i|u\rangle$. If we change the orientation of a vector from right pointing (ket) to left pointing (bra) or vice-versa, we transpose and take the complex conjugate. For example, if $|A\rangle =i\left(\begin{array}{c} 1\\ 0 \end{array}\right)=i|u\rangle$, then $\langle A|=-i\left(\begin{array}{c} 1\;\;0 \end{array}\right)=-i\langle u|$. Therefore, any spin matrix can be written as (+1)|u〉〈u|+(−1)|d〉〈d| where |u〉 and |d〉 are their up and down eigenstates, respectively. A qubit is then constructed from this two-level quantum system, i.e., $|\psi \rangle =c_{1}|u\rangle +c_{2}|d\rangle$ where $|c_{1}{|}^{2}+{\left|c_{2}\right|}^{2}=1$.

An arbitrary spin measurement σ in the $\hat{b}$ direction is given by the spin matrices

(7) $$\sigma =\hat{b}\cdot \vec{\sigma }=b_{x}\sigma_{x}+b_{y}\sigma_{y}+b_{z}\sigma_{z}$$

Again, preparation states |ψ〉 are created from linear combinations of the Pauli spin eigenstates. The average outcome (all we can obtain per NPRF) for a measurement σ on state |ψ〉 is given by

(8) 〈σ〉:=〈ψ|σ|ψ〉

For example, in Figure 1 we have |ψ〉=|u〉 (prepared by the first SG magnets) and $\sigma =\sin\left(\beta \right)\sigma_{x}+\cos\left(\beta \right)\sigma_{z}$ (per the second SG magnets), so 〈σ〉=cos(β) in accord with Equation (1).

Finally, the probability of obtaining a +1 or −1 result for σ is just

(9) $$P\left(+1|\beta \right)=|\langle \psi |\tilde{u}\rangle {|}^{2}=\cos^{2}\left(\frac{\beta }{2}\right)$$

and

(10) $$P\left(-1|\beta \right)=|\langle \psi |\tilde{d}\rangle {|}^{2}=\sin^{2}\left(\frac{\beta }{2}\right)$$

where $|\tilde{u}\rangle$ and $|\tilde{d}\rangle$ are the eigenvectors of σ and $\frac{\beta }{2}$ is the angle between |ψ〉 and $|\tilde{u}\rangle$ in Hilbert space. This agrees with the result from NPRF in Equations (2) and (3). Thus, per Einstein’s definition of a principle theory, “we have an empirically discovered principle that gives rise to mathematically formulated criteria which the separate processes or the theoretical representations of them have to satisfy.”

Again, the Pauli spin matrices are created from the possible measurement outcomes ±1 and the outer products of their eigenstates. Thus, we see that the entire qubit state and measurement structure is operationally self-referential (contextual) in that the preparation states and the measurement operators are not independent. We also see how the principle of NPRF underwrites the QM operational structure for qubits and, therefore, the QIT reconstructions of QM built upon the qubit. In the following, we will review the SU(2)/SO(3) transformation property for qubits via their bipartite entanglement in the Bell spin states.

### 3.1. The Bell Spin States

With that review of the implicit contextuality in the qubit operational formalism and its basis in NPRF, let us explore the conservation being depicted by the Bell spin states and relate it to the correlation function. When considering two-particle states, we will use the juxtaposed notation for our spin states and matrices. Thus, $\sigma_{x}\sigma_{z}|ud\rangle =-|dd\rangle$ and $\sigma_{x}\sigma_{y}|ud\rangle =-i|du\rangle$, for example. Essentially, we are simply ignoring the tensor product sign ⊗, so that $\left(\sigma_{x}⊗\sigma_{z}\right)||u\rangle ⊗|d\rangle \rangle =\sigma_{x}\sigma_{z}|ud\rangle$. It is still easy to see which spin matrix is acting on which Hilbert space vector via the juxtaposition.

The Bell spin states are (again, omitting ⊗)

(11) $$\begin{array}{cc} \; & |\psi_{-}\rangle =\frac{|ud\rangle \;-|du\rangle }{\sqrt{2}}\\ \; & |\psi_{+}\rangle =\frac{|ud\rangle +|du\rangle }{\sqrt{2}}\\ \; & |\phi_{-}\rangle =\frac{|uu\rangle \;-|dd\rangle }{\sqrt{2}}\\ \; & |\phi_{+}\rangle =\frac{|uu\rangle +|dd\rangle }{\sqrt{2}} \end{array}$$

in the eigenbasis of $\sigma_{z}$. The first state $|\psi_{-}\rangle$ is called the “spin singlet state” and it represents a total conserved spin angular momentum of zero (S=0) for the two particles involved. The other three states are called the “spin triplet states” and they each represent a total conserved spin angular momentum of one (S=1, in units of ℏ=1). In all four cases, the entanglement represents the conservation of spin angular momentum for the process creating the state.

**Figure 4:**
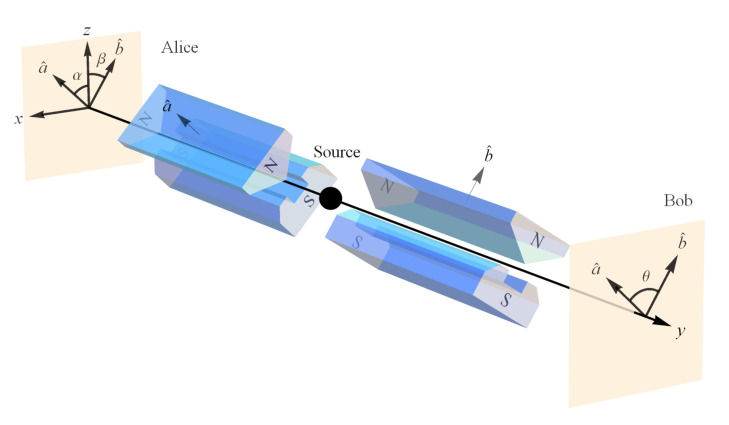
Alice and Bob making spin measurements on a pair of spin-entangled particles with their Stern–Gerlach (SG) magnets and detectors.

Assuming that Alice is making her spin measurement $\sigma_{1}$ in the $\hat{a}$ direction and Bob is making his spin measurement $\sigma_{2}$ in the $\hat{b}$ direction (Figure 4), we have

(12) $$\begin{array}{cc} \; & \sigma_{1}=\hat{a}\cdot \vec{\sigma }=a_{x}\sigma_{x}+a_{y}\sigma_{y}+a_{z}\sigma_{z}\\ \; & \sigma_{2}=\hat{b}\cdot \vec{\sigma }=b_{x}\sigma_{x}+b_{y}\sigma_{y}+b_{z}\sigma_{z} \end{array}$$

Per the formalism explicated above, the correlation functions are given by (again, omitting ⊗)

(13) $$\begin{array}{ccc} \; & \langle \psi_{-}|\sigma_{1}\sigma_{2}|\psi_{-}\rangle = & -a_{x}b_{x}-a_{y}b_{y}-a_{z}b_{z}\\ \; & \langle \psi_{+}|\sigma_{1}\sigma_{2}|\psi_{+}\rangle = & a_{x}b_{x}+a_{y}b_{y}-a_{z}b_{z}\\ \; & \langle \phi_{-}|\sigma_{1}\sigma_{2}|\phi_{-}\rangle = & -a_{x}b_{x}+a_{y}b_{y}+a_{z}b_{z}\\ \; & \langle \phi_{+}|\sigma_{1}\sigma_{2}|\phi_{+}\rangle = & a_{x}b_{x}-a_{y}b_{y}+a_{z}b_{z} \end{array}$$

We now review the conservation being depicted by the Bell spin states, starting with the singlet state $|\psi_{-}\rangle$.

The spin singlet state is invariant under all three SU(2) transformations. For example, $|\psi_{-}\rangle \to |\psi_{-}\rangle$ when we transform our basis per

(14) $$\begin{array}{ccc} |u\rangle & \to & \cos(\Theta )|u\rangle +\sin(\Theta )|d\rangle \\ |d\rangle & \to & -\sin(\Theta )|u\rangle +\cos(\Theta )|d\rangle \end{array}$$

where Θ is an angle in Hilbert space (as opposed to the SG magnet angles in real space). To see what this means in real space, we construct the corresponding spin measurement operator using these transformed up $|\tilde{u}\rangle$ and down $|\tilde{d}\rangle$ vectors

(15) $$|\tilde{u}\rangle \langle \tilde{u}\left|-\right|\tilde{d}\rangle \langle \tilde{d}|=\left(\begin{array}{cc} \cos(2\Theta ) & \sin(2\Theta )\\ \sin(2\Theta ) & -\cos(2\Theta ) \end{array}\right)=\cos\left(2\Theta \right)\sigma_{z}+\sin\left(2\Theta \right)\sigma_{x}$$

Thus, the invariance of the state under this Hilbert space SU(2) transformation means we have rotational (SO(3)) invariance for the SG measurement outcomes in the xz-plane of real space. Specifically, $|\psi_{-}\rangle$ tells us that when the SG magnets are aligned in the *z* direction (Alice and Bob are in the same reference frame) the outcomes are always opposite ($\frac{1}{2}$ of the time ud and $\frac{1}{2}$ of the time du). Since $|\psi_{-}\rangle$ has that same functional form under an SU(2) transformation in Hilbert space representing an SO(3) rotation in the xz-plane per Equations (14) and (15), the outcomes are always opposite ($\frac{1}{2}$ud and $\frac{1}{2}$du) for aligned SG magnets in the xz-plane. That is the “SO(3) conservation” associated with this SU(2) symmetry.

**Figure 5:**
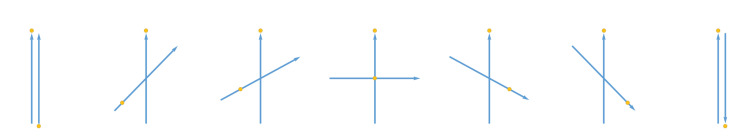
**Average View for the Spin Singlet State**. Reading from left to right, as Bob rotates his SG magnets relative to Alice’s SG magnets for her +1 outcome, the average value of his outcome varies from −1 (totally down, arrow bottom) to 0 to +1 (totally up, arrow tip). This obtains per conservation of spin angular momentum on average in accord with NPRF. Bob can say exactly the same about Alice’s outcomes as she rotates her SG magnets relative to his SG magnets for his +1 outcome. That is, their outcomes can only satisfy conservation of spin angular momentum *on average* in different reference frames, because they only measure ±1, never a fractional result. Thus, just as NPRF in SR leads to a principle explanation of time dilation and Lorentz contraction, we see that NPRF in quantum mechanics (QM) requires quantum outcomes $\pm 1\left(\frac{\hbar }{2}\right)$ for all measurements leading to a principle explanation of Bell state entanglement.

**Figure 6:**
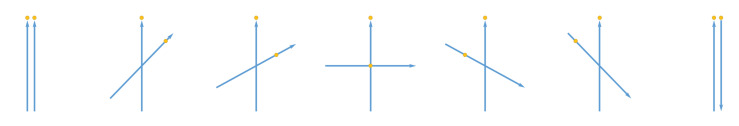
**Average View for the Spin Triplet States**. Reading from the left, as Bob(Alice) rotates his(her) SG magnets relative to Alice’s(Bob’s) SG magnets for her(his) +1 outcome, the average value of his(her) outcome varies from +1 (totally up, arrow tip) to 0 to −1 (totally down, arrow bottom).

Equation (15) shows us that when the angle in Hilbert space is Θ, the angle θ of the rotated SG magnets in the xz-plane is θ=2Θ. The physical reason for this factor of 2 between Θ in Hilbert space and θ in real space can be seen in Figure 5 and Figure 6.

Equation (14) is the Hilbert space SU(2) transformation that represents an SO(3) rotation about the *y* axis in real space and can be written

(16) $$\left(\begin{array}{c} u\\ d \end{array}\right)\to \left(\begin{array}{cc} \cos(\Theta ) & \sin(\Theta )\\ -\sin(\Theta ) & \cos(\Theta ) \end{array}\right)\left(\begin{array}{c} u\\ d \end{array}\right)=\left(\cos\left(\Theta \right)I+i\sin\left(\Theta \right)\sigma_{y}\right)\left(\begin{array}{c} u\\ d \end{array}\right)$$

The SU(2) transformation that represents an SO(3) rotation about the *x* axis in real space can be written

(17) $$\left(\begin{array}{c} u\\ d \end{array}\right)\to \left(\begin{array}{cc} \cos(\Theta ) & i\sin(\Theta )\\ i\sin(\Theta ) & \cos(\Theta ) \end{array}\right)\left(\begin{array}{c} u\\ d \end{array}\right)=\left(\cos\left(\Theta \right)I+i\sin\left(\Theta \right)\sigma_{x}\right)\left(\begin{array}{c} u\\ d \end{array}\right)$$

In addition, the SU(2) transformation that represents an SO(3) rotation about the *z* axis in real space can be written

(18) $$\begin{array}{c} \left(\begin{array}{c} u\\ d \end{array}\right)\to \left(\begin{array}{cc} \cos(\Theta )+i\sin(\Theta ) & 0\\ 0 & \cos(\Theta )-i\sin(\Theta ) \end{array}\right)\left(\begin{array}{c} u\\ d \end{array}\right)=\left(\cos\left(\Theta \right)I+i\sin\left(\Theta \right)\sigma_{z}\right)\left(\begin{array}{c} u\\ d \end{array}\right) \end{array}$$

The invariance of $|\psi_{-}\rangle$ under all three SU(2) transformations is reasonable, since the spin singlet state represents the conservation of a total, directionless spin angular momentum of S=0 and each SU(2) transformation in Hilbert space corresponds to an element of SO(3) in real space. This explains why its correlation function is $-\hat{a}\cdot \hat{b}$, as shown in Equation (13). Now let us look at the spin triplet states.

Starting with $|\phi_{+}\rangle$, the only SU(2) transformation that takes $|\phi_{+}\rangle \to |\phi_{+}\rangle$ is Equation (14). That means this state reflects rotational (SO(3)) invariance for our SG measurement outcomes in the xz-plane. Specifically, $|\phi_{+}\rangle$ means when the SG magnets are aligned in the *z* direction (measurements are being made in the same reference frame) the outcomes are always the same ($\frac{1}{2}$ of the time uu and $\frac{1}{2}$ of the time dd). Since $|\phi_{+}\rangle$ has that same functional form under an SU(2) transformation in Hilbert space representing an SO(3) rotation in the xz-plane per Equations (14) and (15), the outcomes are always the same ($\frac{1}{2}$uu and $\frac{1}{2}$dd) for aligned SG magnets in the xz-plane. That is the “SO(3) conservation” associated with this SU(2) symmetry and it applies only for measurements made at the same angle (in the same reference frame). Here, $|\phi_{+}\rangle$ is only invariant under Equation (14), so we can only expect rotational invariance for our SG measurement outcomes in the xz-plane. This agrees with Equation (13) where we see that the correlation function for arbitrarily oriented $\sigma_{1}$ and $\sigma_{2}$ is $a_{x}b_{x}-a_{y}b_{y}+a_{z}b_{z}$. Therefore, unless we restrict our measurements to the xz-plane, we do not have the rotationally invariant correlation function $\hat{a}\cdot \hat{b}$ as with to the spin singlet state.

For the state $|\phi_{-}\rangle$, we find that the only SU(2) transformation leaving it invariant is Equation (17). Therefore, this state means we have rotational (SO(3)) invariance for the SG measurement outcomes in the yz-plane. Given that $|\phi_{-}\rangle$ is only invariant under Equation (17), we can only expect rotational invariance for our SG measurement outcomes in the yz-plane. This agrees with Equation (13) where we see that the correlation function for arbitrarily oriented $\sigma_{1}$ and $\sigma_{2}$ for $|\phi_{-}\rangle$ is given by $-a_{x}b_{x}+a_{y}b_{y}+a_{z}b_{z}$. So, unless we restrict our measurements to the yz-plane, we do not have the rotationally invariant correlation function $\hat{a}\cdot \hat{b}$ as with the spin singlet state.

Finally, $|\psi_{+}\rangle$ is only invariant under the SU(2) tranformation of Equation (18). Therefore, this state means we have rotational (SO(3)) invariance for our SG measurement outcomes in the xy-plane. However, unlike the situation with $|\psi_{-}\rangle$, we need to transform $|\psi_{+}\rangle$ to either the $\sigma_{x}$ or $\sigma_{y}$ eigenbasis to find the rotationally invariant outcome in the xy-plane. Doing so we find that the outcomes are always the same ($\frac{1}{2}$ of the time uu and $\frac{1}{2}$ of the time dd) in the xy-plane [33]. This agrees with Equation (13) where we see that the correlation function for arbitrarily oriented $\sigma_{1}$ and $\sigma_{2}$ for $|\psi_{+}\rangle$ is given by $a_{x}b_{x}+a_{y}b_{y}-a_{z}b_{z}$. Therefore, unless we restrict our measurements to the xy-plane, we do not have the rotationally invariant correlation function $\hat{a}\cdot \hat{b}$ as with the spin singlet state.

What does all this mean? Obviously, the SU(2) invariance of each of the spin triplet states in Hilbert space represents the SO(3) invariant conservation of spin angular momentum S=1 for each of the planes xz ($|\phi_{+}\rangle$), yz ($|\phi_{-}\rangle$), and xy ($|\psi_{+}\rangle$) in real space. Specifically, when the measurements are being made in the same reference frame (SG magnets are aligned) anywhere in the respective symmetry plane the outcomes are always the same ($\frac{1}{2}$ of the time uu and $\frac{1}{2}$ of the time dd). That is, we have a planar conservation and our experiment would determine the plane. If you want to model a conserved S=1 for some other plane, you simply expand in the spin triplet basis.

With this understanding of the conservation principle at work for entangled qubits, we see why the so-called “mirror quantum mechanics” of Dakic and Brukner [43] does not make sense physically. The “mirror” solution of their reconstruction is regular, but cannot be consistently constructed for systems of three bits. Thus, Dakic and Brukner rule it out for mathematical reasons. We can rule it out already at the level of the two-qubit system because its correlation functions are simply −1 times those in Equation (13). That means the mirror singlet state has total spin angular momentum of 1 instead of zero while the mirror triplet states have total spin angular momentum of zero instead of 1. Thus, the entire structure of rotational invariance shown above for standard QM, which makes sense physically, because S=0 is directionless while S=1 is directional, becomes nonsense physically in “mirror quantum mechanics.”

In conclusion, we point out that the conservation at work here deals with the measurement outcomes proper. Per Dakic and Brukner’s axiomatic reconstruction of quantum theory [43], the Bell spin states represent measurement outcomes on an entangled pair of “elementary systems,” and “An elementary system has the information carrying capacity of at most one bit.” Thus, the measurement outcomes do not represent the observed part of some hidden information carried by an underlying quantum system. Colloquially put, Alice and Bob’s measurement outcomes constitute all of the available information.

### 3.2. NPRF and the Bell State Correlation Function

We now extrapolate our understanding of the qubit Hilbert space structure that follows from NPRF to the correlation functions for the Bell spin states of entangled qubit pairs. Assuming only that Alice and Bob each measure +1 and −1 with equal frequency at any arbitrary settings α and β, respectively (NPRF), the correlation function is [32,33]

(19) $$\langle \alpha ,\beta \rangle =\frac{1}{2}{\left(+1\right)}_{A}\bar{BA+}+\frac{1}{2}{\left(-1\right)}_{A}\bar{BA-}$$

where $\bar{BA+}$ is the average of Bob’s outcomes when Alice measured +1 (denoted ${\left(+1\right)}_{A}$) and $\bar{BA-}$ is the average of Bob’s outcomes when Alice measured −1 (denoted ${\left(-1\right)}_{A}$). That is, we have partitioned the data per Alice’s equivalence relation, i.e., Alice’s +1 results and Alice’s −1 results. Note that this correlation function is independent of the formalism of QM, all we have assumed is that Alice and Bob each measure +1 and −1 with equal frequency for all measurement settings per NPRF. We now analyze the situation from Alice’s perspective.

We will explain the case of the spin triplet state, as the case of the spin singlet state is analogous [33] (Figure 5 and Figure 6). As with the single-particle state, classical intuition leads us to expect the projection of the spin angular momentum vector of Alice’s particle ${\vec{S}}_{A}=+1\hat{a}$ along $\hat{b}$ is ${\vec{S}}_{A}\cdot \hat{b}=+\cos\left(\theta \right)$ where again θ is the angle between the unit vectors $\hat{a}$ and $\hat{b}$ (Figure 4). Again, this is because the prediction from classical physics is that all values between $+1\left(\frac{\hbar }{2}\right)$ and $-1\left(\frac{\hbar }{2}\right)$ are possible outcomes for a measurement of angular momentum. According to Alice, had Bob measured at her angle, i.e., oriented his SG magnets in the same direction, he would have found the spin angular momentum vector of his particle was ${\vec{S}}_{B}={\vec{S}}_{A}=+1\hat{a}$ per conservation of spin angular momentum. Since he did not measure the spin angular momentum of his particle in her reference frame (same angle), he should have obtained a projected fraction of the length of ${\vec{S}}_{B}$, i.e., ${\vec{S}}_{B}\cdot \hat{b}=+1\hat{a}\cdot \hat{b}=\cos\left(\theta \right)$ (Figure 2). But according to NPRF, Bob only ever obtains +1 or −1 just like Alice, so he cannot measure the required fractional outcome to explicitly conserve spin angular momentum per Alice. Therefore, as with the single-particle case, NPRF means that Bob’s outcomes must satisfy “average-only” projection (Figure 3 and Figure 6), which means

(20) $$\bar{BA+}=\cos\left(\theta \right)$$

Given this constraint per NPRF, as with the single-particle case, we can now use NPRF to find the joint probabilities for Alice and Bob’s outcome pairs. Looking at Table 1, the rows and columns all sum to $\frac{1}{2}$ because both Alice and Bob must observe +1 half of the time and −1 half of the time per NPRF, which also asserts that the table is symmetric so that P(−1,+1∣θ)=P(+1,−1∣θ). The average of Bob’s outcomes given that Alice observes a +1 is

(21) $$\bar{BA+}=2P\left(+1,+1|\theta \right)\left(+1\right)+2P\left(+1,-1|\theta \right)\left(-1\right)=\cos\left(\theta \right)$$

using conservation per NPRF. Together with the constraints on the rows/columns

$$\begin{array}{cc} P(+1,+1|\theta )+P(+1,-1|\theta ) & =\frac{1}{2}\\ P(+1,-1|\theta )+P(-1,-1|\theta ) & =\frac{1}{2}, \end{array}$$

we can uniquely solve for the joint probabilities

(22) $$P\left(+1,+1|\theta \right)=P\left(-1,-1|\theta \right)=\frac{1}{2}\cos^{2}\left(\frac{\theta }{2}\right)$$

and

(23) $$P\left(+1,-1|\theta \right)=P\left(-1,+1|\theta \right)=\frac{1}{2}\sin^{2}\left(\frac{\theta }{2}\right).$$

Now we can use these to compute $\bar{BA-}$

(24) $$\bar{BA-}=2P\left(-1,+1|\theta \right)\left(+1\right)+2P\left(-1,-1|\theta \right)\left(-1\right)=-\cos\left(\theta \right)$$

Using Equations (21) and (24) in Equation (19) we obtain

(25) $$\langle \alpha ,\beta \rangle =\frac{1}{2}{\left(+1\right)}_{A}\left(\cos\left(\theta \right)\right)+\frac{1}{2}{\left(-1\right)}_{A}\left(-\cos\left(\theta \right)\right)=\cos\left(\theta \right)$$

which is precisely the correlation function for a spin triplet state in its symmetry plane found in Section 3.1.

Of course, Bob could partition the data according to his equivalence relation, i.e., his reference frame, so that it is Alice who must average her results, as obtained in her reference frame, to conserve spin angular momentum. Thus, the mathematical structure is again consistent with NPRF. In addition, this symmetry in perspectives requiring that Alice and Bob measure ±1 with equal frequency for all settings, plus the average-only nature of the correlations, is precisely what precludes signalling, regardless of whether Alice’s measurement settings and outcomes are spacelike or timelike related to Bob’s.

**Figure 7:**
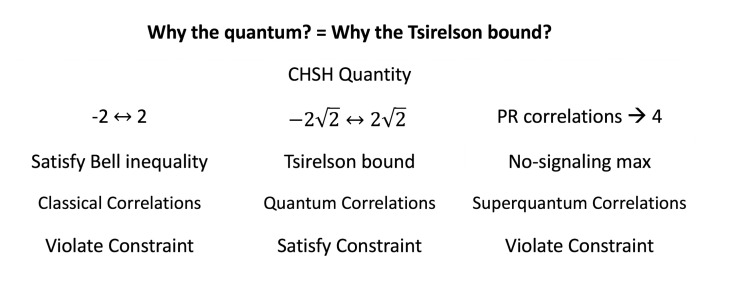
The “constraint” is conservation per no preferred reference frame.

Finally, since it is precisely this correlation function that is responsible for the Tsirelson bound [60,61,62], we see that NPRF is ultimately responsible for the Tsirelson bound. This answers Bub’s question, “why is the world quantum and not classical, and why is it quantum rather than superquantum, i.e., why the Tsirelson bound for quantum correlations?” [53,63,64] (Figure 7). This also tells us why higher-dimensional generalized probability theories are not realized in Nature, i.e., the conservation principle for the fundamental two-bit system must be a qubit to accord with NPRF.

## 4. Discussion

We have offered a principle account of EPR correlations (quantum entanglement) and quantum contextuality by applying a generalization of the relativity principle (“no preferred reference frame,” NPRF) to the measurement of Planck’s constant *h* to underwrite the qubit Hilbert space structure with its SU(2)/SO(3) transformation properties. That is, the qubit structure is the foundation of “Hilbert space as a projective geometry (i.e., the subspace structure of Hilbert space)” whence the EPR correlations. In doing so, we see that NPRF is to Hardy and Dakic and Brukner’s reconstructions of QM, as NPRF is to the Lorentz transformations of SR, since the postulates of SR can be stated as NPRF applied to the measurement of the speed of light *c*. This answers Allori’s question cited earlier, “Should I understand SSM’s view as a principle theory? (But if so, which are the principles?)” [31] (p. 344).

Conservation per NPRF then accounts for no-signalling and the violations of the Bell inequality precisely to the Tsirelson bound [32], which explains why so-called “superquantum correlations” [65] and higher-dimensional generalized probability theories are not realized in Nature. Conservation per NPRF also shows so-called “mirror quantum mechanics” to be nonphysical already at the level of the two-qubit system. Thus, besides revealing a deep unity between SR and QM (Table 2), NPRF resolves many quantum mysteries.

This certainly is not what QIT had in mind for their reconstruction project. That is, they intended their reconstructions of QM would be to the “standard axioms of [quantum theory]” as “Einstein’s postulates of SR are to the Lorentz transformations.” As things stand now, there is no obvious connection between the interpretation-project of QM and the QIT-project [66]. In this regard, keep in mind that the postulates of SR are about the physical world in spacetime; thus, in keeping with this analogy, QIT must eventually make such correspondence to reach their lofty goals and escape the clever, but inherent instrumentalism of standard QM. Our principle account of QM, whereby NPRF is to the QIT reconstructions of QM as NPRF is to the Lorentz transformations of SR, precisely addresses the need for QIT to make correspondence with phenomena in spacetime. While these QM reconstructions do not account for all of quantum phenomena, they certainly cover bipartite qubit entanglement whence the mysteries of quantum entanglement and quantum contextuality. But, most importantly, QM reconstructions built upon the qubit Hilbert space structure explicate the essential mathematical framework for rendering QM a principle theory via NPRF.

The general idea here is that in order to make progress in the foundations of QM and in unifying QM and SR, we cannot merely continue to provide constructive empirically equivalent interpretations that lead neither to new predictions, new unifying insights, nor to underwriting QM itself. This is what we are attempting to do here. It may seem a bit counterintuitive that NPRF underwrites both SR and QM, since quantum entanglement has been alleged by some to imply faster-than-light influences contra SR [67,68,69]. Per Popescu and Rohrlich [65]:

Quantum mechanics, which does not allow us to transmit signals faster than light, preserves relativistic causality. But quantum mechanics does not always allow us to consider distant systems as separate, as Einstein assumed. The failure of Einstein separability violates, not the letter, but the spirit of special relativity, and left many physicists (including Bell) deeply unsettled.

Obviously QM (non-relativistic quantum mechanics) is not Lorentz invariant, so it certainly differs from SR in that regard. QM follows from Lorentz invariant quantum field theory only in the low energy approximation [70] (p. 173). However, claiming that SR and QM are somehow at odds based on quantum entanglement has empirical consequences, because we have experimental evidence verifying the violation of the Bell inequality in accord with quantum entanglement. Thus, if the violation of the Bell inequality is problematic for SR, then SR is being empirically challenged in some sense, hence Bell’s unease.

For example, Newtonian mechanics deviates from SR because it is not Lorentz invariant. Accordingly, Newtonian mechanics predicts that velocities add in a different fashion than in SR, so imagine we found experimentally that velocities add according to Newtonian mechanics. That would not only mean Newtonian mechanics and SR are at odds, that would mean SR has been empirically refuted. Bell’s unease aside, clearly, few people believe that QM has literally falsified SR. But we have gone further to show that not only is there no tension between QM and SR in substance or spirit, NPRF provides a completely local principle account of EPR correlations. Indeed, even the no-signalling feature of entangled qubits follows necessarily from NPRF. Thus, far from being incompatible, SR and QM share a deep coherence via NPRF (Table 2). This principle explanation for EPR correlations requires no violation of the causal structure of SR and it does not require the addition of a preferred frame as some non-local interpretations do, such as Bohmian mechanics and spontaneous collapse interpretations. Furthermore, this principle explanation for EPR correlations requires no causal or constructive explanation whatsoever, and that includes retrocausal mechanisms and processes. Indeed, this principle account of QM does not even require a metaphysical commitment to the block universe.

Despite the fact that this principle explanation supplies a unifying framework for both QM and SR, some might demand a constructive explanation with its corresponding “knowledge of how things in the world work, that is, of the mechanisms (often hidden) that produce the phenomena we want to understand” [71] (p. 15). This is “the causal/mechanical view of scientific explanation” per Salmon [71] (p. 15). Thus, as with SR, not everyone will consider our principle account of QM to be explanatory since, “By its very nature such a theory-of-principle explanation will have nothing to say about the reality behind the phenomenon” [72] (p. 331). As Lorentz famously complained about SR [73] (p. 230):

Einstein simply postulates what we have deduced, with some difficulty and not altogether satisfactorily, from the fundamental equations of the electromagnetic field.

And, Albert Michelson said [74]:

It must be admitted, these experiments are not sufficient to justify the hypothesis of an ether. But then, how can the negative result be explained?

In other words, neither was convinced that NPRF was sufficient to explain time dilation and Lorentz contraction. More recently Brown has made a similar claim [46] (p. 76):

What has been shown is that rods and clocks must behave in quite particular ways in order for the two postulates to be true together. But this hardly amounts to an explanation of such behaviour. Rather things go the other way around. It is because rods and clocks behave as they do, in a way that is consistent with the relativity principle, that light is measured to have the same speed in each inertial frame.

In other words, the assumption is that the true or fundamental “explanation” of EPR correlations must be a constructive one in the sense of adverting to causal processes or causal mechanisms. Apparently for people with such a Reichenbachian or constructive mind-set, any principle explanation must be accounted for by some such story, e.g., the luminiferous ether. Indeed, contrary to all accepted physics, Brown and Pooley [46] have recently called for such a constructive explanation even in SR. Brown and Pooley like to make this a debate about constructive versus “geometric” explanation. They believe that the principle explanation of Lorentz contractions in SR is underwritten only by the geometry of Minkowski spacetime.

We think this misses the point, as one could believe that SR provides a principle explanation of Lorentz contractions without being a realist or a substantivialist about Minkowski spacetime. Notice, there is nothing inherently geometric about our principle explanation of EPR correlations in particular, or of NPRF in general. We would say that Brown and Pooley got it exactly wrong. It is QM that needs to become explicitly more like SR, not the other way around. Indeed, as noted in Section 2, neither textbook QM nor any of its constructive interpretations, ever made for very convincing constructive theories anyway. If QM had struck people as being like statistical mechanics, there would be no cottage industry of cooking up constructive interpretations and no need for anything like QIT reconstructions. We hope to have shed some light on why QM actually works as it does.

After Einstein published SR in 1905, physicists gradually lost interest in theories of the luminiferous ether, preferred reference frames, or any other causal account of Lorentz contractions and time dilation. Even Lorentz seemed to acknowledge the value of this principle explanation when he wrote [73] (p. 230):

By doing so, [Einstein] may certainly take credit for making us see in the negative result of experiments like those of Michelson, Rayleigh, and Brace, not a fortuitous compensation of opposing effects but the manifestation of a general and fundamental principle.

Thus, 85 years after the publication of the EPR paper without a consensus constructive or causal account of EPR correlations, perhaps it is time to consider the possibility that physicists will eventually likewise stop looking for constructive accounts of EPR correlations. After all, we now know that the widely accepted relativity principle is precisely the principle that resolves a plethora of QM mysteries. And, as Pauli once stated [75] (p. 33):

‘Understanding’ nature surely means taking a close look at its connections, being certain of its inner workings. Such knowledge cannot be gained by understanding an isolated phenomenon or a single group of phenomena, even if one discovers some order in them. It comes from the recognition that a wealth of experiential *facts are interconnected and can therefore be reduced to a common principle*. In that case, certainty rests precisely on this wealth of facts. The danger of making mistakes is the smaller, the richer and more complex the phenomena are, and the simpler is the common principle to which they can all be brought back. ... ‘Understanding’ probably means nothing more than having whatever ideas and concepts are needed to recognize that a great many different phenomena are part of a coherent whole. [Italics ours.]

One could hardly ask for a “simple common principle” or “one clear, simple sentence” more compelling than “no preferred reference frame” to convey “the central point and its necessity in the construction of the world.” Perhaps causal accounts of quantum entanglement and quantum contextuality are destined to share the same fate as theories of the luminiferous ether. Perhaps this principle account will finally cause us to let go of the Reichenbachian past, and go back to the future with Einstein’s insights about principle explanation.

**Postscript**

We understand how deep the causal and dynamical explanatory bias goes and thus we know that people will feel like we’ve dodged something important by not saying more about our ontology/beables and more about what the role of the wavefunction is on our account. That is, our answer to the challenge to causality by the EPR correlations resides in the probability structure of QM alone, “the kinematic part of QM” per Bub [55], so we have not offered anything concerning QM dynamics. And of course when people demand to know your “ontology,” they mean that in the constructive, causal and dynamical sense of the word. Part of the causal and dynamical bias, the very reason many people think constructive explanation must be fundamental, is because they assume the world is composed of or otherwise determined by such beables. That is, they want to know what the world is made of or what matter is. Herein, we will briefly provide our answers to those questions by explaining how our principle account of QM is a realist, psi-epistemic account. Let us note however that one need not share all our commitments herein to find our preceding principle account compelling.

Let us begin with the psi-epistemic part. Explaining what this means for us requires going beyond the scope of this paper, as the question of Schrödinger dynamics differs from the question of the probability structure of QM [8,22]. If one constructs the differential equation (Schrödinger equation) corresponding to the Feynman path integral, the time-dependent foliation of spacetime gives the wavefunction ψ(x,t) in concert with our time-evolved perceptions and the fact that we do not know when the outcome is going to occur. Once one has an outcome, both the configuration $x_{o}$, that is the specific spatial locations of the experimental outcomes, and time $t_{o}$ of the outcomes are fixed, so the wavefunction ψ(x,t) of configuration space becomes a probability amplitude $\psi (x_{o},t_{o})$ in spacetime, i.e., a probability amplitude for a specific outcome in spacetime. Again, the evolution of the wavefunction in configuration space before it becomes a probability amplitude in spacetime is governed by the Schrödinger equation.

However, the abrupt change from wavefunction in configuration space to probability amplitude in spacetime is not governed by the Schrödinger equation. In fact, if the Schrödinger equation is universally valid, it would simply say that the process of measurement should entangle the measurement device with the particle being measured, leaving them both to evolve according to the Schrödinger equation in a more complex configuration space (as in the relative-states formalism shown below). Certain interpretations of QM notwithstanding, we do not seem to experience such entangled existence in configuration space, which would contain all possible experimental outcomes. Instead, we experience a single experimental outcome in spacetime.

This contradiction between theory and experience is called the “measurement problem.” However, the time-evolved story in configuration space is not an issue with the path integral formalism as we interpret it, because we compute $\psi (x_{o},t_{o})$ directly. That is, in asking about a specific outcome we must specify the future boundary conditions that already contain definite and unique outcomes. Thus, the measurement problem is a non-starter for us. When a QM interpretation assumes the wavefunction is an epistemological tool rather than an ontological entity, that interpretation is called “psi-epistemic.” In our path integral, contextuality-based account the wavefunction in configuration space is not even used, so our account is trivially psi-epistemic. Thus, our account of the wavefunction is very much like Rovelli’s as we described it earlier in the paper. Our view is a complete rejection of Hilbert space realism and the like. In short, we would say that the operational recipe of textbook Hamiltonian-based QM with its Schrödinger dynamics, is from the ant’s-eye perspective, the very best one could do given that the primary goal is prediction of the temporal evolution of the QM state, regardless of its ultimate ontic status.

This covers the psi-epistemic part of our view, but what about the realist part? Of course, again, this begs the question of what is required for a realist account of QM. Let us begin with the obvious. Unless one is begging the question, there is nothing inherently anti-realist or subjectivist about principle explanation. After all, NPRF generally, and “average-only” conservation specifically, are real, mind-independent and perspective-independent facts about spacetime. Indeed, by this measure, our principle explanation for EPR correlations and the like, is much more realist than the retrocausal perspectival causal explanations and also more realist than most of the psi-epistemic accounts on offer. This bears repeating. What is doing the explanatory work on our principle account of QM, just as with SR, are mind and perspective-independent facts about the world.

Again, one might nonetheless feel that our account fails to be realist because it does not provide a specific constructive ontology, such as particles, fields or waves. The first thing to note here is that the whole point of principle explanation is that it is compatible with any number of such ontologies, and that it is not incumbent upon the purveyor of such explanations to provide a constructive ontology, because such an ontology is not relevant to the explanation at hand. Indeed, as we have pointed out on numerous occasions, our principle explanation of EPR correlations is even compatible with unmediated exchanges/direct action between source and detector, i.e., the idea that there are no worldlines of counterfactual definiteness that connect the source and the detector. But we get it, many people will feel that something is missing if we cannot say exactly, in constructive terms, what goes on between source and detector. From their perspective, so far, the only thing we have told you is that the wavefunction and Hilbert space are not real, but not, what is “real.” Is it particles, fields, waves in spacetime, or something else?

This is not an easy question to answer for many reasons. First, as is well known, the standard definitions that provide the essence of “particles,” “waves” and “fields” are all violated by one or another weirdness of QM, e.g., that particles are strictly point like, that fields are a fully continuous and contiguous medium with all definite values at every point in spacetime for which counterfactual definiteness obtains, and that waves (in spacetime at least) must be fully and always wave-like in their behavior and be instantiated in some material or energetic medium. Second, if contextuality is a fundamental fact about the world as seems to be the case based on experimental evidence and several theorems, as we noted earlier, this calls into question the very idea of classical objects composed of or realized by autonomous self-existent QM entities with definite, intrinsic properties, and what Einstein called “primitive thisness,” or what is sometimes called haecceity.

In accord with Rovelli’s relational QM, our conjecture, given all of the above, is that the search for such a fundamental context-free ontology is misguided. Indeed, both relational QM and our view are inspired by the lessons of SR, that certain facts, entities and quantities are reference frame dependent, and we also attempt to apply that idea to QM. There are certainly other similarities as well, e.g., the idea that QM is complete, the psi-epistemic take, the focus on information, and the invocation of contextuality. However, in order to explain the EPR correlations, relational QM merely defers to the entangled qubit Hilbert space structure, while QIT note that the mystery is, “Why the qubit structure? Why not classical bits? Or, generalized higher-dimensional bits? The no-signalling requirement does not suffice to rule them out.” And, that answer is not to be found in the conservation represented by the Bell spin states when the measurements are made in the same reference frame (SG magnets are at the same angle). Those perfect correlations are easily replicated by assuming “hidden, definite values.’’ The answer resides in the conservation represented by the Bell spin states when the measurements are made in *different* reference frames (SG magnets are at different angles). It is there that one finds “average-only’’ conservation per NPRF due to “average-only’’ projection per NPRF, as explained in Section 3. By taking seriously NPRF and not just relationalism, we have underwritten QM and explained its informational structure without giving up The Absoluteness of Observed Events, as is entailed by relational QM in certain cases. NPRF and The Absoluteness of Observed Events is the very heart of SR, and the very basis for its relationalism. Our diagnosis is that Rovelli drops this insight because unlike explanation in SR, he has not fully transcended the dynamical and causal explanatory bias.

As for fundamental ontology, we would say that multiscale contextuality itself is fundamental, and sometimes, depending on various contextual features, reality (whatever its ultimate *metaphysical* nature), behaves in a particle-like, field-like or wave-like fashion. We think the twin-slit experiment alone is sufficient to see how this might be so. While all this is beyond the scope of our paper, in our view, environmental decoherence, so called QM non-separability, so called QM holism, so called QM relationalism, QM dispositionalism, etc., are really just symptomatic of the fundamentality of multiscale contextuality. And furthermore, while the contextuality in question is often manifested in dynamical and causal interactions, the deeper contextuality that explains and underwrites certain aspects of those interactions is sometimes non-causal, non-dynamical and spatiotemporal–what we call adynamical global constraints. For example, as we demonstrated herein, the kind of contextuality we see in the case of EPR correlations is a consequence of NPRF. We see nothing inherently anti-realist about this view, as again, conservation laws and multiscale contextuality are real mind and perspective-independent facts about the world. What QM and relativity are really telling us is that to exist, i.e., to be a diachronic entity in space and time, is to interact with the rest of the universe creating a consistent, shared set of classical information constituting the universe [8,34]. Again, this is another way in which our view is a fully realist one, given that NPRF is at the core of our account of the physical world [8,34], as with SR itself, The Absoluteness of Observed Events can never be violated on our view.

## Figures and Tables

**Table 1:** **Joint probabilities for Alice and Bob’s outcome pairs for the entangled particle experiment in Figure 4.** The table is symmetric due to NPRF.

		Bob		
		+1	−1	Total
Alice	+1	P(+1,+1 ∣θ)	P(+1,−1 ∣θ)	1/2
	−1	P(+1,−1 ∣θ)	P(−1,−1 ∣θ)	1/2
	Total	1/2	1/2	1

**Table 2:** **Comparing special relativity with quantum mechanics according to no preferred reference frame (NPRF)**. Because Alice and Bob both measure the same speed of light *c*, regardless of their motion relative to the source per NPRF, Alice (Bob) may claim that Bob’s (Alice’s) length and time measurements are erroneous and need to be corrected (Lorentz contraction and time dilation). Likewise, because Alice and Bob both measure the same values for spin angular momentum ±1$\left(\frac{\hbar }{2}\right)$, regardless of their SG magnet orientation relative to the source per NPRF, Alice (Bob) may claim that Bob’s (Alice’s) individual ±1 values are erroneous and need to be corrected (averaged, Figure 3, Figure 5 and Figure 6). In both cases, NPRF resolves the mystery it creates. In SR, the apparently inconsistent results can be reconciled via the relativity of simultaneity. That is, Alice and Bob each partition spacetime per their own equivalence relations (per their own reference frames), so that equivalence classes are their own surfaces of simultaneity and these partitions are equally valid per NPRF. This is completely analogous to QM, where the apparently inconsistent results per the Bell spin states arising because of NPRF can be reconciled by NPRF via the “relativity of data partition.” That is, Alice and Bob each partition the data per their own equivalence relations (per their own reference frames), so that equivalence classes are their own +1 and −1 data events and these partitions are equally valid per NPRF.

Special Relativity	Quantum Mechanics
Empirical Fact: Alice and Bob both measure *c*,	Empirical Fact: Alice and Bob both measure $\pm 1\left(\frac{\hbar }{2}\right)$,
regardless of their motion relative to the source	regardless of their SG orientation relative to the source
Alice(Bob) says of Bob(Alice): Must correct his(her)	Alice(Bob) says of Bob(Alice): Must average his(her)
length and time measurements	±1 outcomes for projection/conservation
NPRF: Relativity of simultaneity	NPRF: Relativity of data partition

## Data Availability

Not applicable.
